# Leprosy incidence in the state of Piauí, Brazil: spatial distribution and temporal trend, 2013–2023

**DOI:** 10.1590/1806-9282.20260739

**Published:** 2026-10-30

**Authors:** Manuela Solyano Almeida de Oliveira, Ana Beatriz Oliveira Guimarães, Maria Antônia de Jesus Sousa, Leticia Sampaio de Andrade, Victor Augusto da Paz, Mikaelle Rodrigues Gomes, Ana Lúria Liriel de Sousa Lima, João Gabriel Noleto Ferreira de Matos, Államy Danilo Moura e Silva

**Affiliations:** 1 Centro Universitário Santo Agostinho – Teresina (PI), Brazil.; 2 Universidade de Salvador – Salvador (BA), Brazil.

**Keywords:** Leprosy, Epidemiology, Time factors, Spatial analysis, Public health surveillance

## Abstract

**OBJECTIVE:**

The aim of this study was to analyze the incidence, spatial distribution, and temporal trend of leprosy in Piauí, Brazil, between 2013 and 2023.

**METHODS:**

This ecological study used secondary data on new leprosy cases from the Notifiable Diseases Information System, available through DATASUS/TABNET. Cases among adults aged 18 years or older living in Piauí were included. For the spatial analysis, cases were grouped by health macro-region and development region of residence, and absolute and relative frequencies were calculated to identify territories with the highest concentration of notifications. Temporal trends were assessed by simple linear regression, with a 5% significance level.

**RESULTS:**

From 2013 to 2023, Piauí recorded 12,256 new leprosy cases. The highest concentration occurred in the Meio Norte macro-region and the Entre Rios development region. A significant decreasing trend was observed in Piauí (β=-54.11; 95%CI -85.24 to -22.98; p=0.003) and Brazil (β=-1,361.24; 95%CI -2,151.01 to -571.46; p=0.004), with a marked reduction in notifications in 2020. Low educational attainment, lack of bacilloscopy testing, and dimorphic clinical form predominated.

**CONCLUSION:**

Leprosy remains a relevant public health problem in Piauí, with spatial concentration and signs compatible with delayed diagnosis and surveillance weaknesses. Strengthening early detection, active surveillance, and health education is necessary to reduce transmission.

## INTRODUCTION

Leprosy is a chronic infectious disease caused by Mycobacterium leprae, which primarily affects the skin and peripheral nerves. When not diagnosed and treated early, it can lead to physical disabilities, social stigma, and long-term functional impairment^1^.

Despite the availability of multidrug therapy and the expansion of primary health care, leprosy remains a public health challenge in Brazil, with persistent regional inequalities and higher burden in historically vulnerable areas, especially the North, Northeast, and Midwest regions^2,3^.

In Piauí, previous studies have shown high endemicity, spatial clusters, and operational weaknesses in diagnosis and follow-up, indicating the need for updated analyses after the COVID-19 pandemic period^4^.

Spatial distribution and time-series analyses contribute to identifying territories with persistent transmission, monitoring the impact of health service disruptions, and supporting public health planning. Therefore, this study aimed to analyze the incidence, spatial distribution, and temporal trend of leprosy in Piauí, Brazil, between 2013 and 2023^5,6^.

## METHODS

This was an ecological, analytical study with descriptive spatial and time-series components. The study followed the general principles recommended for reporting observational studies^7^.

The study setting was the state of Piauí, located in Northeastern Brazil. Piauí comprises 224 municipalities, four health macro-regions, and 12 development regions. According to the Brazilian Institute of Geography and Statistics, the state has more than 3.2 million inhabitants and marked internal demographic heterogeneity^8^.

The study population included all new leprosy cases recorded among adults aged 18 years or older living in Piauí from 2013 to 2023. A census approach was used. Records outside the period or outside the geographic scope were excluded.

Data were obtained from the Notifiable Diseases Information System (SINAN), accessed through DATASUS/TABNET, using aggregated and publicly available records^9^.

The variables analyzed included year of notification, region of residence, Brazilian region, state, capital city, Piauí health macro-region, Piauí development region, bacilloscopy at notification, reactional episode, schooling, clinical form, and number of new cases.

Data were organized in Microsoft Excel 365 and analyzed in IBM SPSS Statistics, version 26.0. Descriptive statistics were presented as absolute (n) and relative (%) frequencies. For the spatial analysis, new cases were grouped according to the predefined territorial units available in the database: the four health macro-regions and 12 development regions of Piauí, based on region of residence. The absolute number and relative proportion of cases were calculated for each territorial unit, allowing comparison of the geographic concentration of notifications across the state. Temporal trends were assessed using simple linear regression, with year of notification as the independent variable and the number of new cases as the dependent variable. The parameters estimated were Pearson's correlation coefficient (r), coefficient of determination (R^2^), regression coefficient (β), 95%CI, and p-value. A 5% significance level was adopted. A positive β with p<0.05 indicated an increasing trend, a negative β with p<0.05 indicated a decreasing trend, and p≥0.05 indicated stability.

Ethics committee approval was waived because the study used aggregated, publicly accessible, secondary data without individual identification. The study complied with Brazilian ethical standards for research using public databases^10^.

## RESULTS

From 2013 to 2023, 357,904 new leprosy cases were recorded in Brazil. The Northeast accounted for the largest absolute number of notifications (152,524), followed by the Midwest (75,032), North (66,971), Southeast (51,572), and South (11,805). In the same period, Piauí recorded 12,256 new cases in the state-specific tabulations and 12,093 cases in the regional comparison, reflecting the use of different DATASUS filters.

Regarding diagnostic, clinical, and sociodemographic characteristics in the national database, bacilloscopy was most frequently recorded as not performed (32.4%), followed by negative (29.2%) and positive results (24.9%). Ignored or blank bacilloscopy information accounted for 13.4% of records. Most cases were reported without a reactional episode (62.0%), but 23.8% of this field was not recorded. Low schooling was frequent, especially incomplete 1st to 4th grade (19.6%), and ignored or blank schooling data also represented a substantial proportion (19.4%). The dimorphic clinical form predominated (48.7%), followed by the virchowian (18.0%), tuberculoid (12.3%), and indeterminate (11.6%) forms (Table 1).

**Table 1:** Diagnostic, clinical, and sociodemographic characteristics of new leprosy cases at notification, Brazil, 2013–2023.

Variable	Category	n	%
Bacilloscopy at notification	Ignored or blank	48,002	13.4
Bacilloscopy at notification	Positive	89,297	24.9
Bacilloscopy at notification	Negative	104,633	29.2
Bacilloscopy at notification	Not performed	115,972	32.4
Reactional episode	Not recorded	85,076	23.8
Reactional episode	Type 1 reaction	34,731	9.7
Reactional episode	Type 2 reaction	11,309	3.2
Reactional episode	Type 1 and 2 reactions	5,015	1.4
Reactional episode	No reaction	221,773	62.0
Schooling	Ignored or blank	69,298	19.4
Schooling	Illiterate	30,919	8.6
Schooling	Incomplete 1st–4th grade	70,175	19.6
Schooling	Completed 4th grade	25,676	7.2
Schooling	Incomplete 5th–8th grade	51,836	14.5
Schooling	Completed elementary school	21,555	6.0
Schooling	Incomplete high school	22,714	6.3
Schooling	Completed high school	46,122	12.9
Schooling	Incomplete higher education	5,048	1.4
Schooling	Completed higher education	12,319	3.4
Schooling	Not applicable	2,242	0.6
Clinical form	Ignored or blank	12,299	3.4
Clinical form	Indeterminate	41,470	11.6
Clinical form	Tuberculoid	43,897	12.3
Clinical form	Dimorphic	174,395	48.7
Clinical form	Virchowian	64,346	18.0
Clinical form	Unclassified	21,497	6.0

Source: Brazilian Ministry of Health, SVS; SINAN Net. Note: The totals in this table refer to Brazil (n=357,904), as extracted from the national DATASUS/TABNET tabulation. Percentages may not total exactly 100.0% because of rounding.

In Piauí, the distribution of cases was heterogeneous across health macro-regions (Table 2). The Meio Norte macro-region concentrated 6,447 cases, corresponding to 52.6% of all new cases in the state. Cerrados accounted for 2,261 cases (18.4%), followed by the Semi-Arid macro-region with 1,826 cases (14.9%) and the Coastal macro-region with 1,722 cases (14.1%).

**Table 2:** Distribution of new leprosy cases by health macro-region, Piauí, Brazil, 2013–2023.

Health macro-region	2013	2014	2015	2016	2017	2018	2019	2020	2021	2022	2023	Total
Semi-Arid	177	207	228	152	189	189	156	101	115	134	124	1,826
Meio Norte	672	703	604	583	734	691	632	364	446	484	447	6,447
Coastal	153	138	159	192	187	183	163	91	156	168	110	1,722
Cerrados	248	296	267	189	223	224	170	146	161	142	157	2,261
Total	1,250	1,344	1,258	1,116	1,333	1,287	1,121	702	878	928	838	12,256

Source: Brazilian Ministry of Health, SVS; SINAN Net. Note: Values are absolute numbers of new cases. The original regional names were maintained when appropriate.

The analysis by development region showed even greater spatial concentration (Table 3). Entre Rios accounted for 5,797 cases, representing 47.3% of the state total. The second highest total occurred in Vale dos Rios Piauí e Itaueiras (912; 7.4%), followed by Cocais (862; 7.0%), Planície Litorânea (860; 7.0%), Vale do Rio Guaribas (769; 6.3%), Chapada das Mangabeiras (706; 5.8%), and Carnaubais (650; 5.3%). The lowest absolute totals were observed in Tabuleiros do Alto Parnaíba, Vale do Sambito, Vale do Canindé, Chapada Vale do Rio Itaim, and Serra da Capivara.

**Table 3:** Distribution of new leprosy cases by development region, Piauí, Brazil, 2013–2023.

Development region	2013	2014	2015	2016	2017	2018	2019	2020	2021	2022	2023	Total
Carnaubais	70	35	47	55	60	51	44	39	56	104	78	650
Chapada das Mangabeiras	73	99	95	57	76	84	51	41	45	29	41	706
Cocais	77	76	71	73	100	116	90	46	63	82	56	862
Entre Rios	602	668	557	528	674	640	588	325	390	380	369	5,797
Planície Litorânea	76	62	88	119	87	67	73	45	93	86	54	860
Serra da Capivara	45	49	47	36	39	58	41	44	36	32	42	477
Tabuleiros do Alto Parnaíba	27	23	19	13	11	15	13	11	13	9	11	166
Vale do Canindé	37	39	48	35	52	40	34	17	26	14	17	365
Vale do Rio Guaribas	77	99	84	65	80	66	64	46	49	66	49	769
Vale do Sambito	30	37	42	26	32	18	15	19	16	25	20	285
Vale dos Rios Piauí e Itaueiras	103	125	106	83	97	67	65	50	67	72	63	912
Chapada Vale do Rio Itaim	33	32	54	26	25	65	43	19	24	29	38	407
Total	1,250	1,344	1,258	1,116	1,333	1,287	1,121	702	878	928	838	12,256

Source: Brazilian Ministry of Health, SVS; SINAN Net. Note: Values are absolute numbers of new cases.

Simple linear regression showed a statistically significant decreasing trend in Piauí (β=-54.11; 95%CI -85.24 to -22.98; p=0.003) and in Brazil (β=-1,361.24; 95%CI -2,151.01 to -571.46; p=0.004). Significant reductions were also observed in the South, Southeast, North, and Northeast regions, whereas the Midwest showed a non-significant reduction and was therefore classified as stable.

Among Brazilian capitals, 77,785 new cases were recorded. Teresina reported 4,700 cases, while the highest absolute totals occurred in São Luís (8,387), Fortaleza (8,358), Recife (6,937), Palmas (5,331), and Cuiabá (5,096). A marked reduction in notifications was observed in 2020 in almost all capitals, followed by a partial recovery from 2021 onward.

The annual pattern was similar across macro-regions and development regions: relative stability from 2013 to 2019, a pronounced reduction in 2020, persistently lower counts in 2021, a modest increase in 2022, and a new decrease in 2023. This pattern suggests that the pandemic period may have affected diagnosis, access to services, and notification capacity.

## DISCUSSION

The concentration of cases in the Meio Norte macro-region and in the Entre Rios development region indicates a heterogeneous spatial pattern. This finding is consistent with previous evidence from Piauí, which identified hyperendemic areas and persistent concentration in territories that include the state capital and areas with greater health service availability^4^. The high number of cases in Teresina may therefore reflect both transmission intensity and greater diagnostic and notification capacity.

The temporal analysis showed a statistically significant reduction in new leprosy cases in Piauí between 2013 and 2023. This finding should be interpreted with caution because the sharp reduction in 2020 coincided with the COVID-19 pandemic, when access to health services, active case finding, and routine surveillance activities were substantially disrupted^1,2,11,12^.

The predominance of cases in historically endemic Brazilian regions, particularly the Northeast, North, and Midwest, reinforces the persistence of regional inequalities in leprosy burden^2,3^. These inequalities are associated with social vulnerability, barriers to timely diagnosis, stigma, and unequal access to health services. In this context, leprosy should be interpreted not only as an infectious disease but also as a marker of structural inequities.

The frequent occurrence of dimorphic and virchowian clinical forms in the national dataset is relevant because these forms are commonly associated with multibacillary disease, longer periods of transmissibility, and delayed diagnosis. The high proportion of nonperformed bacilloscopy and incomplete information on reactional episodes also indicates operational weaknesses that may compromise clinical management, epidemiological monitoring, and the quality of health information systems^2,4^.

The reduction in notifications in 2020 is unlikely to represent a true abrupt decline in transmission. Studies conducted during the pandemic reported changes in the organization of primary care, reduced access to consultations and laboratory tests, interruptions in educational activities, and difficulties in monitoring contacts^11,12^. These disruptions may have led to undernotification and delayed diagnosis, with possible future increases in disability at diagnosis.

The findings support the need to strengthen integrated public policies focused on early detection, active surveillance, contact examination, health education, and improvement of information completeness. Priority should be given to territories with greater concentration of cases, especially Meio Norte and Entre Rios, while maintaining surveillance in regions with lower absolute numbers but possible underdetection.

This study has limitations. The analysis was based on secondary data, which are subject to underreporting, incomplete fields, and changes in notification practices. In addition, the spatial analysis was descriptive and based on absolute case counts and proportions by predefined territorial units, without adjustment for population size or assessment of spatial autocorrelation; therefore, comparisons between territories should be interpreted cautiously. Even so, the study provides an updated overview of the spatial distribution and temporal trend of leprosy in Piauí and identifies priority areas for public health action.

## CONCLUSION

Leprosy remains a relevant public health problem in Piauí. The spatial concentration of cases in Meio Norte and Entre Rios, together with evidence of delayed diagnosis and incomplete surveillance records, indicates territorial inequalities that require targeted surveillance actions. Although a statistically significant decrease in new cases was observed from 2013 to 2023, the sharp reduction in 2020 suggests possible undernotification related to the COVID-19 pandemic. Strengthening integrated public policies, active case detection, health education, and surveillance quality is necessary to reduce transmission and support progress toward elimination.

## Data Availability

The data analyzed in this study are publicly available through DATASUS/TABNET and SINAN. The datasets used in the analyses are also available from the corresponding author upon reasonable request.
